# Development and Validation of an Individualized Nomogram to Identify Undifferentiated‐Predominant Mixed‐Type Early Gastric Cancer

**DOI:** 10.1111/1751-2980.70047

**Published:** 2026-04-28

**Authors:** Lin Lin Shao, Yu Miao Zheng, Qian Zhang, Chuan Guo Guo, Peng Li

**Affiliations:** ^1^ Department of Gastroenterology, Beijing Friendship Hospital Capital Medical University Beijing China; ^2^ National Clinical Research Center for Digestive Disease, Beijing Key Laboratory for Precancerous Lesion of Digestive Disease Beijing Digestive Disease Center Beijing China

**Keywords:** clinicopathological feature, endoscopic feature, *Helicobacter pylori*, nomogram, undifferentiated‐type‐predominant mixed‐type early gastric cancer

## Abstract

**Objectives:**

Diagnosis of undifferentiated‐type‐predominant mixed‐type early gastric cancer (UM‐EGC) remains challenging due to its complex histological features and overlapping characteristics with other gastric cancer types. We aimed to develop a novel nomogram based on clinicopathological and endoscopic features and validate its performance in predicting UM‐EGC prior to endoscopic treatment.

**Methods:**

In this retrospective single‐center study, 808 patients with early gastric cancer (EGC) who underwent curative endoscopic submucosal dissection were included. Among them, 493 were assigned to the training cohort and 84 to the external validation cohort. Clinicopathological characteristics and endoscopic features were compared between differentiated EGC and UM‐EGC using logistic regression analysis. A predictive nomogram was constructed and evaluated.

**Results:**

Multivariable regression analysis identified open‐type atrophic gastritis (O1–O3) (odds ratio [OR] 0.25, 95% confidence interval [CI] 0.08–0.82), IIb (OR 9.72, 95% CI 3.01–31.35), IIc (OR 7.75, 95% CI 2.81–21.39), discolored lesion (OR 4.12, 95% CI 1.57–10.80), horizontal location at the greater curvature (OR 2.98, 95% CI 1.15–7.75) or anterior wall (OR 2.91, 95% CI 1.26–6.74), and previous *H. pylori* eradication (OR 0.23, 95% CI 0.09–0.55) as independently associated with UM‐EGC. UM‐EGC was also more susceptible to metachronous cancer (OR 5.50, 95% CI 1.30–23.21). The nomogram demonstrated good discriminative ability with an area under the receiver operating characteristic curve of 0.83 (95% CI 0.77–0.87) in the training cohort and 0.82 (95% CI 0.69–0.98) in the external validation cohort.

**Conclusion:**

This nomogram comprising clinicopathological and endoscopic features may assist in the preoperative prediction of UM‐EGC risk.

**Trial Registration:**

The clinical trial registration number for patient source in this study is ChiCTR1800017117

## Graphical abstract

This study identified open‐type atrophic gastritis, flat or depressed morphology, faded appearance, tumor location on the greater curvature or anterior wall, xanthoma, and previous *Helicobacter pylori* eradication as independent risk factors for undifferentiated‐type‐predominant mixed‐type early gastric cancer (UM‐EGC). Based on these features, we constructed and validated a practical nomogram that can assist endoscopists in the timely and accurate diagnosis of UM‐EGC, thereby informing appropriate treatment selection. CAG, chronic atrophic gastritis; D‐EGC, differentiated early gastric cancer; EGC, early gastric cancer; ESD, endoscopic submucosal dissection; ROC, receiver operating characteristic.

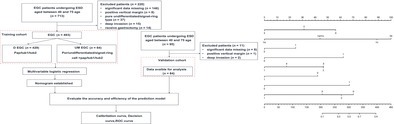

## 1 Introduction

Gastric cancer (GC) is one of the most common malignancies worldwide, ranking the fifth most frequently diagnosed cancer and the fourth leading cause of cancer‐related deaths globally in 2020, leading to a major health burden worldwide [1]. Similarly, GC accounts for the top five new cancer cases and mortality in China [2]. Thanks to the advancement of endoscopic techniques, gastroscopy enables the identification and timely intervention of early gastric cancer (EGC), which has significantly improved patient outcomes. In addition, the introduction of endoscopic submucosal dissection (ESD) has revolutionized the minimally invasive treatment of EGC [3].

ESD allows en bloc resection of the tumor in clinical practice; therefore, it is widely used to treat differentiated early gastric cancer (D‐EGC), which has a low risk of lymph node metastasis. While surgery or gastrectomy with lymph node dissection used to be the gold standard for curative treatment of undifferentiated early gastric cancer (UD‐EGC), which primarily consists of signet ring cell carcinoma, mucinous adenocarcinoma, and poorly differentiated adenocarcinoma, due to the high risks of lymph node metastasis and poor prognosis. Remarkable progress has been made for the treatment of UD‐EGC, and the indications for endoscopic treatment of UD‐EGC have been expanded. According to the 5th edition of the Japanese Gastric Cancer Treatment Guidelines [4], UD‐EGC has negligible risk of lymph node metastasis when reaching 20 mm or less in size and in the absence of ulceration or lymphovascular involvement. However, previous studies have demonstrated that curative resection cannot be achieved with ESD for UD‐EGC with expanded indications, and the rate of non‐curative resection ranges from 14.6% to 51.5% [5, 6]. Notably, GC tissues are often histologically heterogeneous and do not always have one single histological type of cancerous cells but contain a mixture of several different cell types. Mixed‐type GC can be classified into differentiated‐type‐predominate mixed‐type and undifferentiated‐type‐predominate mixed‐type based on its main component [7, 8]. Recent studies have reported that mixed‐type cancer has a high risk of lymph node metastasis [9, 10]. In the presence of an undifferentiated component, treatment of mixed‐type GC needs to be cautious. Therefore, identifying patients with undifferentiated‐type‐predominate mixed‐type early gastric cancer (UM‐EGC) during endoscopy is vital for the selection of treatment strategies.

As the precise diagnosis of UM‐EGC remains challenging in clinical practice due to its complex histological features and overlapping characteristics with other GC subtypes, in this study we aimed to compare the clinicopathological characteristics and endoscopic features of UM‐EGC with those of D‐EGC which were curatively resected by ESD, and to identify UM‐EGC patients who were treated with ESD through the development and validation of a nomogram.

## 2 Patients and Methods

### 2.1 Patient Enrollment

In this single‐center retrospective study, patients with EGC aged between 40 and 75 years who underwent ESD at the Department of Gastroenterology, Beijing Friendship Hospital, Capital Medical University (Beijing, China) from January 2016 to June 2024 were initially screened for eligibility. The patients were recruited from both the Xicheng and Tongzhou campuses of the hospital. All diagnostic procedures, inclusion/exclusion criteria, and pathological assessments were standardized in both campuses. Patients from the Xicheng campus, enrolling mainly those from the urban areas of Beijing, were recruited as the training cohort. Among them, 220 patients were excluded due to significant missing clinical or pathological data for analysis (*n* = 146), positive vertical resection margins (*n* = 8), ambiguous or unclassified histological type (*n* = 37), deep submucosal invasion (*n* = 15), and those who subsequently received gastrectomy (*n* = 14). Finally, the training cohort comprised 493 patients with EGC, including 429 patients with D‐EGC (high and moderately differentiated EGC) and 64 patients with UM‐EGC, respectively. In addition, the validation cohort consisted of 84 patients with EGC who underwent ESD between 2018 and 2022 at the Tongzhou campus, which predominantly serves residents of the Tongzhou District and the neighboring Hebei Province, comprising 7 UM‐EGC cases and 77 D‐EGC cases.

**FIGURE 1:**
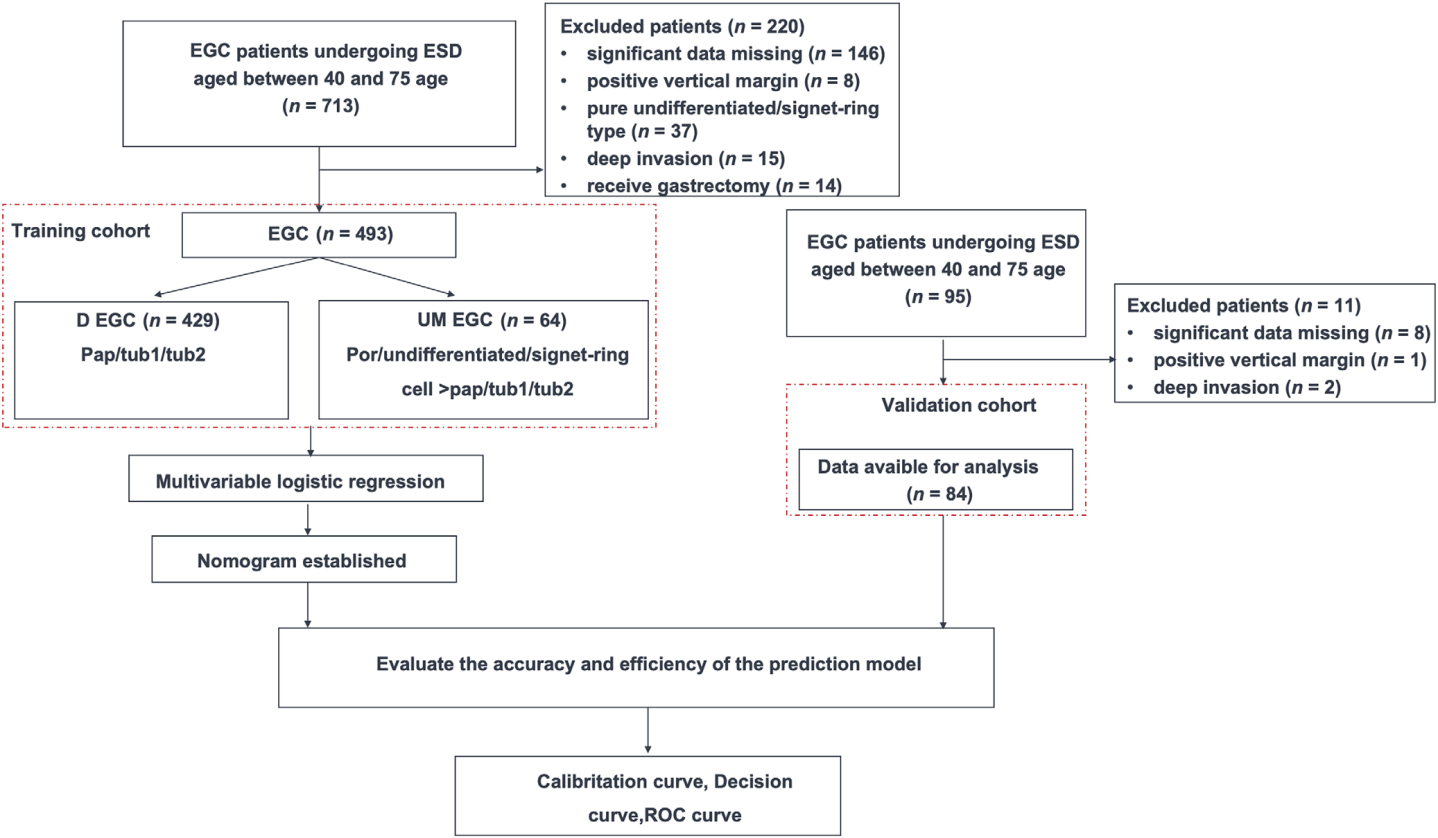
Flowchart of the patient selection process. D‐EGC, differentiated early gastric cancer; EGC, early gastric cancer; ESD, endoscopic submucosal dissection; UM‐EGC, undifferentiated‐type‐predominant mixed‐type early gastric cancer; pap, papillary adenocarcinoma; tub1, well‐differentiated tubular adenocarcinoma; tub2, moderately differentiated tubular adenocarcinoma; ROC, receiver operating characteristic.

All lesions resected by ESD were evaluated by pathologists specialized in gastroenterology. Only patients having lesions with the depth of invasion limited to the submucosal layer, with negative horizontal and vertical margins, were included; while those who had undergone chemotherapy and/or radiation therapy or received prior ESD or surgery were excluded from the analysis. The flowchart showing patient inclusion and exclusion is presented in Figure 1. This study was conducted in accordance to the Declaration of Helsinki (Brazil, 2013), and was approved by the Scientific Research Ethical Licensing Committee of the Beijing Friendship Hospital, Capital Medical University (2018‐P2‐058‐01). Written informed consent was obtained from all participants before the ESD procedure.

### 2.2 Clinicopathological Evaluation

Clinicopathological characteristics of the patients as well as endoscopic and pathological findings of the lesions were extracted from the Health Information System and recorded for analysis, which included patients' age, sex, smoking status, alcohol consumption, comorbid hypertension, diabetes mellitus, hyperlipidemia, family history of GC, and previous *Helicobacter pylori* infection and treatment. Tumor location, depth of invasion, coloration, ulceration, xanthoma, and the background mucosa were extracted from their pathological and endoscopic reports. *H. pylori* infection in GC and the adjacent non‐cancerous tissues was also reviewed. Atrophic gastritis was diagnosed as closed (C1, C2, C3) and open (O1, O2, O3) types according to the Kimura–Takemoto classification [11].

For each ESD specimen, sections were prepared at 3‐mm intervals for pathological evaluation. When two or more lesions were identified, they were measured and assessed based on the lesion with the maximum diameter or depth of invasion. Depth of invasion was defined as the distance from the lowest level of the muscularis mucosa (or surface of ulceration) to the deepest point of tumor invasion. The level of submucosal invasion was divided into two subtypes, namely, mucosa and submucosa (SM), and the latter was further classified into upper third (SM1), middle third (SM2), and lower third (SM3). Ulceration was defined histologically as a disruption of the muscularis mucosa with or without granulation tissue formation or submucosal fibrosis. In addition, metachronous gastric carcinoma was defined as a new‐onset GC detected in a different location from the primary ESD site, which occurred at least 6 months after the initial ESD procedure.

### 2.3 Statistical Analysis

Categorical variables were expressed as numbers and percentages or frequencies, and the differences in these variables between groups were compared by using Chi‐square test. Univariable and multivariable logistic regression analyses were then performed to investigate the association between the identified candidate variables and the histological type of EGC, and odds ratio (OR) and 95% confidence interval (CI) were calculated. Variables with a *p* value of less than 0.05 in univariable analysis were further included in the multivariable model. In addition, variables with strong clinical relevance, as well as categorical variables for which any subcategory showed statistical significance (*p* < 0.05) in the univariable analysis, were also retained in the multivariable model, regardless of the overall *p* value. To assess the adequacy of the sample size for our primary findings, a post‐hoc power calculation was performed for the multivariate logistic regression model. The events‐per‐variable (EPV) ratio was calculated as the number of UM‐EGC events divided by the number of predictor variables included in the final multivariable logistic regression model. In the current study, the outcome was observed in 64 patients, and the final model contained eight variables. Thus, the EPV was approximately 8.

A nomogram including predictors with statistical significance for UM‐EGC, as identified from the multivariable analysis, was constructed in the training cohort. The receiver operating characteristic (ROC) curve for the nomogram was applied, and the area under the ROC curve (AUC) was calculated. Meanwhile, decision curve analysis (DCA) was used to assess the net benefit of the nomogram in the clinical setting. One thousand bootstrapping resamples in the training cohort were then used for internal validation. The nomogram was also validated in the validation cohort and was assessed by AUC and DCA. The Brier score was also calculated to assess the performance of the nomogram.

All statistical analyses were performed by using SPSS Statistics version 24.0 (IBM, New York, Armonk, USA) and R version 4.4.1 (R Foundation, Vienna, Austria). *p* < 0.05 was set as statistically significant.

## 3 Results

### 3.1 Clinicopathological and Endoscopic Characteristics Between Different Histological Types of EGC

**FIGURE 2:**
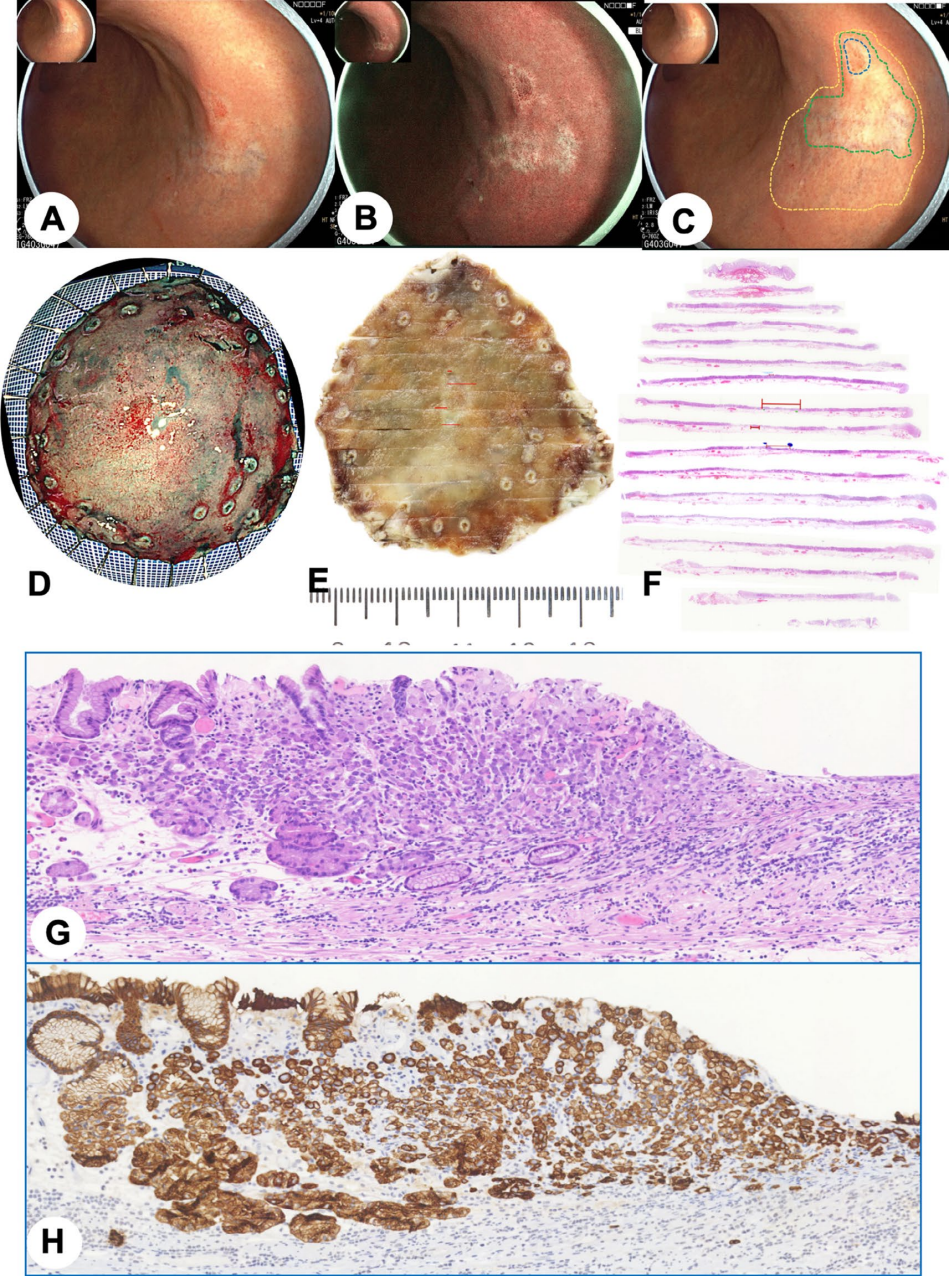
Endoscopic and pathological features of an undifferentiated‐type‐predominant mixed‐type early gastric cancer (UM‐EGC) case with challenging diagnosis. (A) White‐light imaging (WLI) showing a flat, faded lesion at the posterior wall of the corpus. (B) Narrow‐band imaging showing a relatively clear border of the lesion. (C) WLI showing the lesion consisting of three parts: the carcinoma (blue), atrophic gastritis (green), and atrophic gastritis (yellow). (D) Macroscopic image of endoscopic submucosal dissection (ESD)‐resected specimen of the lesion. (E) Formalin‐fixed and paraffin‐embedded specimen; red lines show the cancer lesion. (F) Histological imaging of the carcinoma (HE stain; red line). (G) Microscopic imaging of the lesion consisting of undifferentiated and signet‐ring cell carcinoma (HE stain, ×40). (H) CK staining showing the signet‐ring cell carcinoma component (CK stain, ×40).

Among the 713 patients who received ESD for EGC in the training cohort, 493 patients were finally included. Their clinicopathological characteristics are summarized in Table 1. Among all these EGC cases in the training set, 429 (87.0%) patients at a median age of 65 years were diagnosed with D‐EGC, and the other 64 (13.0%) were diagnosed as having UM‐EGC. There were no differences in patients' age, sex, alcohol consumption, smoking status, hypertension, hyperlipidemia, diabetes mellitus, or a family history of GC between the D‐EGC and UM‐EGC subgroups. However, there was a significant divergence in *H. pylori* infection rate (*p* = 0.001). The rate of chronic atrophic gastritis, as identified under endoscopy, was more prevalent in the D‐EGC subgroup (90.9% vs. 81.3%, *p* = 0.035). In addition, flat or depressed macroscopic tumor type (*p* < 0.001) and faded lesion (*p* = 0.002) were more common in cases with UM‐EGC. Over half (55.9%) the D‐EGC was most commonly located in the lower third of the stomach, whereas UM‐EGC was more prevalent in the middle third of the stomach (42.2%) (*p* = 0.007). In addition, UM‐EGC showed a markedly higher rate of submucosal invasion (26.6% vs. 7.7%, *p* < 0.001). Interestingly, metachronous carcinoma was more commonly observed in UM‐EGC (12.5% vs. 1.4%, *p* < 0.001), while lesion multiplicity, and ulceration did not differ between D‐EGC and UM‐EGC cases. Representative images of UM‐EGC are shown in Figure 2.

**TABLE 1:** Clinicopathological characteristics and endoscopic features of differentiated early gastric cancer (D‐EGC) and undifferentiated‐type‐predominant mixed‐type early gastric cancer (UM‐EGC) in the training cohort.

Characteristics	Overall (*N* = 493)	D‐EGC (*n* = 429)	UM‐EGC (*n* = 64)	*p* value
Age (*n*, %)				
≤ 60 years	155 (31.4)	131 (30.5)	24 (37.5)	0.330
> 60 years	338 (68.6)	298 (69.5)	40 (62.5)	
Sex (*n*, %)				
Male	344 (69.8)	305 (71.1)	39 (60.9)	0.132
Female	149 (30.2)	124 (28.9)	25 (39.1)	
Atrophic gastritis (*n*, %)				
No	51 (10.3)	39 (9.1)	12 (18.8)	0.035
Closed type (C1–C3)	348 (70.6)	304 (70.9)	44 (68.8)	
Open type (O1–O3)	94 (19.1)	86 (20.0)	8 (12.5)	
Multiple lesions (*n*, %)	122 (24.7)	110 (25.6)	12 (18.8)	0.300
Macroscopic type (*n*, %)				
IIa	132 (26.8)	126 (29.4)	6 (9.4)	< 0.001
IIa + IIc	157 (31.8)	143 (33.3)	14 (21.9)	
IIb	60 (12.2)	47 (11.0)	13 (20.3)	
IIc	144 (29.2)	113 (26.3)	31 (48.4)	
White light color, faded (*n*, %)	40 (8.1)	28 (6.5)	12 (18.8)	0.002
Ulceration (*n*, %)	50 (10.1)	44 (10.3)	6 (9.4)	1.000
Vertical location (*n*, %)				
Upper third	96 (19.5)	83 (19.3)	13 (20.3)	0.007
Middle third	133 (27.0)	106 (24.7)	27 (42.2)	
Lower third	264 (53.5)	240 (55.9)	24 (37.5)	
Horizontal location (*n*, %)				
Lesser curvature	178 (36.1)	164 (38.2)	14 (21.9)	0.011
Greater curvature	68 (13.8)	57 (13.3)	11 (17.2)	
Anterior wall	92 (18.7)	72 (16.8)	20 (31.2)	
Posterior wall	155 (31.4)	136 (31.7)	19 (29.7)	
Depth of invasion (*n*, %)				
Mucosal	443 (89.9)	396 (92.3)	47 (73.4)	< 0.001
Submucosal	50 (10.1)	33 (7.7)	17 (26.6)	
Metachronous carcinoma (*n*, %)	14 (2.8)	6 (1.4)	8 (12.5)	< 0.001
Xanthoma (*n*, %)	58 (11.8)	56 (13.1)	2 (3.1)	0.036
Hypertension (*n*, %)	205 (41.6)	178 (41.5)	27 (42.2)	1.000
Diabetes mellitus (*n*, %)	94 (19.1)	77 (17.9)	17 (26.6)	0.143
Hyperlipidemia (*n*, %)	191 (38.7)	162 (37.8)	29 (45.3)	0.308
Smoking (*n*, %)	195 (39.6)	171 (39.9)	24 (37.5)	0.823
Alcohol consumption (*n*, %)	151 (30.6)	131 (30.5)	20 (31.2)	1.000
Family history of gastric cancer (*n*, %)	122 (24.7)	112 (26.1)	10 (15.6)	0.097
*Helicobacter pylori* infection (*n*, %)				
Current	135 (27.4)	107 (24.9)	28 (43.8)	0.001
Eradicated	162 (32.9)	153 (35.7)	9 (14.1)	
Negative	62 (12.6)	51 (11.9)	11 (17.2)	
Unclear	134 (27.2)	118 (27.5)	16 (25.0)	

### 3.2 Univariable and Multivariable Logistic Regression Analyses of Predictors for UM‐EGC Risk

Univariable and multivariable logistic regression analyses of the predictors for UM‐EGC risk are listed in Table 2. A flat type (IIb) (OR 9.72, 95% CI 3.01–31.35, *p* < 0.001) or depressed (IIc) tumor type (OR 7.75, 95% CI 2.81–21.39, *p* < 0.001), faded lesion (OR 4.12, 95% CI 1.57–10.80, *p* = 0.004), tumor location at the greater curvature of the stomach (OR 2.98, 95% CI 1.15–7.75, *p* = 0.025) or anterior wall (OR 2.91, 95% CI 1.26–6.74, *p* = 0.013), and the presence of metachronous carcinoma (OR 5.50, 95% CI 1.30–23.21, *p* = 0.020) were associated with an increased risk of UM‐EGC, while open‐type chronic atrophic gastritis (O1–O3) (OR 0.25, 95% CI 0.08–0.82, *p* = 0.023) and previous *H. pylori* eradication (OR 0.23, 95% CI 0.09–0.55, *p* = 0.001) were associated with a decreased risk of UM‐EGC.

**TABLE 2:** Univariable and multivariable logistic regression analyses of predictors for undifferentiated‐type‐predominant mixed‐type early gastric cancer (UM‐EGC) risk.

Variables	Univariable analysis			Multivariable analysis		
	OR	95% CI	*p* value	OR	95% CI	*p* value
Age (years)						
≤ 60	1.00 (reference)					
> 60	0.73	0.42–1.27	0.264			
Sex						
Male	1.00 (reference)			1.00 (reference)		
Female	1.58	0.92–2.72	0.101	1.67	0.86–3.11	0.133
Atrophy						
No	1.00 (reference)			1.00 (reference)		
Closed (C1‐C3)	0.47	0.23–0.97	0.040	0.51	0.21–1.24	0.139
Open (O1‐O3)	0.30	0.11–0.80	0.016	0.25	0.08–0.82	0.023
Macroscopic type						
IIa	1.00 (reference)			1.00 (reference)		
IIa + IIc	2.06	0.77–5.51	0.152	2.90	0.99–8.51	0.051
IIb	5.81	2.09–16.17	0.001	9.72	3.01–31.35	< 0.001
IIc	5.76	2.32–14.32	< 0.001	7.75	2.81–21.39	< 0.001
White light color						
Red	1.00 (reference)			1.00 (reference)		
Faded	3.31	1.58–6.90	0.001	4.12	1.57–10.80	0.004
Ulceration	0.91	0.37–2.22	0.828			
Vertical location						
Upper third	1.00 (reference)					
Middle third	1.63	0.79–3.35	0.186			
Lower third	0.64	0.31–1.31	0.221			
Horizontal location						
Lesser curvature	1.00 (reference)			1.00 (reference)		
Greater curvature	2.26	0.97–5.26	0.059	2.98	1.15–7.75	0.025
Anterior wall	3.25	1.56–6.80	0.002	2.91	1.26–6.74	0.013
Posterior wall	1.64	0.79–3.39	0.184	1.54	0.68–3.48	0.297
Metachronous carcinoma	8.66	2.81–26.67	< 0.001	5.50	1.30–23.21	0.020
Xanthoma	0.22	0.05–0.90	0.034	0.25	0.05–1.16	0.076
Diabetes mellitus	1.65	0.90–3.03	0.104			
Family history of gastric cancer	0.52	0.26–1.06	0.074	0.66	0.30–1.44	0.300
*Helicobacter pylori* infection						
Current	1.00 (reference)			1.00 (reference)		
Eradicated	0.23	0.10–0.50	< 0.001	0.23	0.09–0.55	0.001
Negative	0.82	0.38–1.79	0.624	0.90	0.36–2.27	0.827
Unclear	0.52	0.27–1.01	0.054	0.60	0.28–1.27	0.182

Abbreviations: CI, confidence interval; OR, odds ratio.

To validate the robustness of our findings, we conducted a post‐hoc power analysis based on the primary positive finding of faded appearance, and found the calculated statistical power for detecting this independent predictor of UM‐EGC to be 0.91.

### 3.3 Nomogram of UM‐EGC Development and Validation

**FIGURE 3:**
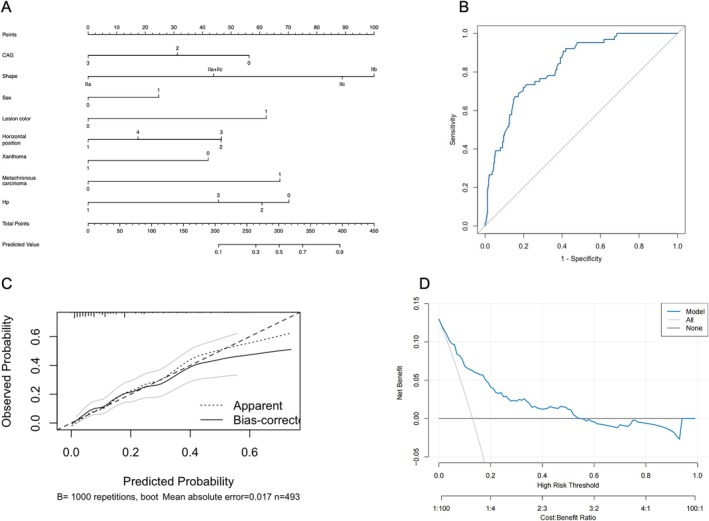
Development and performance of (A) the nomogram conducted based on clinicopathological factors and endoscopic features in the training set according to (B) the receiver operating characteristic (ROC) curve, (C) calibration curve, and (D) decision curve analysis. CAG, chronic atrophic gastritis; Hp, *Helicobacter pylori* .

Based on the predictors identified from the multivariable regression analysis, we developed a nomogram to individualize the prediction of UM‐EGC (Figure 3A), which was used to calculate a score sum that corresponded to a patient's specific probability of having UM‐EGC. In the training set, the nomogram demonstrated strong discriminatory power, with an AUC of 0.83 (95% CI 0.77–0.87) (Figure 3B), along with moderate sensitivity (70%, 95% CI 64%–86%) and high specificity (82%, 95% CI 70%–79%). Internal validation using 1000 bootstrap resamples confirmed a good calibration, indicating close agreement between predictions and observations (Figure 3C). Moreover, DCA further established the clinical utility of the nomogram, showing a superior net benefit across a wide range of risk thresholds (Figure 3D).

### 3.4 External Validation of the Nomogram

**FIGURE 4:**
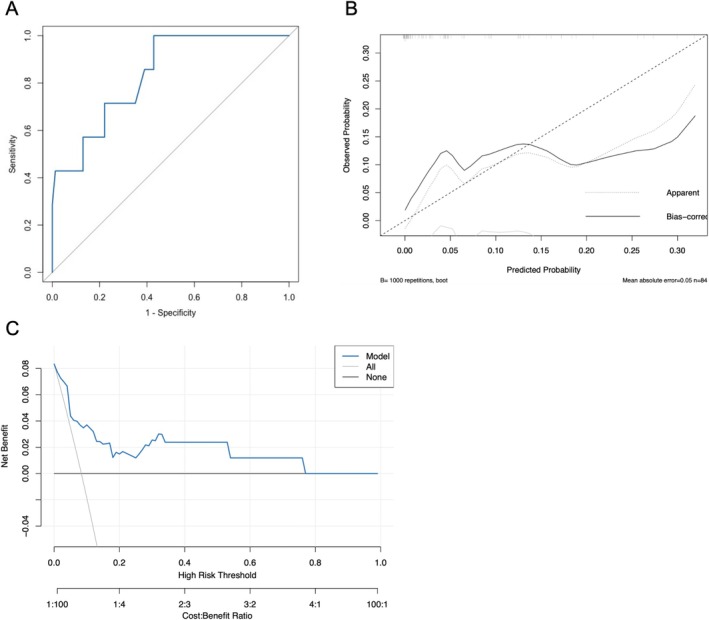
Performance of the nomogram in the validation set according to (A) the receiver operating characteristic curve, (B) calibration curve, and (C) decision curve analysis.

A comparison of baseline characteristics between the training and validation cohorts is provided in Table S1, showing that the training and validation cohorts were generally comparable in terms of age, sex, lesion location and depth of invasion, atrophic gastritis, *H. pylori* infection, and family history of GC (all *p* > 0.05). However, statistically significant differences were observed in lesion multiplicity, lesion macroscopic type, and lesion color (*p* < 0.05). Despite these differences, the performance of the nomogram was robustly established in the validation cohort of 84 EGC patients, yielding an AUC of 0.82 (95% CI 0.69–0.98) (Figure 4A). Bootstrap validation in this cohort resulted in a Brier score of 0.06, suggesting a slight tendency to underestimate the risk (Figure 4B). Nevertheless, DCA confirmed that the nomogram maintained a favorable net benefit for clinical decision‐making compared to alternative strategies (Figure 4C). Also, to assess the internal validity, we performed fivefold cross‐validation, which yielded an AUC of 0.73 (95% CI 0.63–0.82) and per‐fold AUC of 0.80, 0.79, 0.65, 0.63, and 0.76.

## 4 Discussion

Accurate distinction between histological subtypes of EGC is critical for determining treatment strategies, particularly for endoscopic resection. Among these, mixed‐type GC—characterized by coexisting differentiated and undifferentiated components—has a poorer prognosis compared with purely differentiated or undifferentiated types. In this study, we compared the clinicopathological characteristics and endoscopic features between D‐EGC and UM‐EGC, and developed a simple, clinically applicable prediction model for UM‐EGC, which was subsequently validated in an external cohort. This model showed a moderate sensitivity (70%) and a high specificity (82%), offering a practical tool to support potential treatment decision‐making in clinical setting.

In the training cohort of 493 patients, we identified significant differences in several endoscopic and clinicopathological features between D‐EGC and UM‐EGC, including the presence of chronic atrophic gastritis, macroscopic type, faded appearance, tumor location, and depth of invasion. Consistent with prior studies, elevated lesions were more frequent in D‐EGC. Metachronous carcinoma was more common in UM‐EGC, aligning with previous studies that associated undifferentiated histology with a higher risk of metachronous lesions [12]. Furthermore, *H. pylori* infection status differed significantly between the groups [13, 14]. While D‐EGC is known to undergo morphological changes after eradication therapy that can mimic gastritis and complicate endoscopic diagnosis, the characteristics of UM‐EGC appear less influenced by *H. pylori* [15, 16, 17]. Meanwhile, Tanaka et al. [18] found that the clinicopathological characteristics were similar between *H. pylori* ‐infected and *H. pylori* ‐eradicated patients with UM‐EGC. A recent study showed that the prevalence of *H. pylori* ‐negative GC was 2.0%–10.6% of the general Japanese population [19], including undifferentiated GC, fundic GC, and other types. Thus, the relationship between histological subtype and *H. pylori* infection—including post‐eradication and infection‐negative status—remains complex and warrants further investigation.

Given that advanced imaging techniques like magnifying narrow‐band imaging have shown limited advantage for diagnosing flat/depressed UM‐EGC [20], combining routine endoscopic findings with clinicopathological features may help accurate identification of the components of the lesions. Notable alternative models include the deep learning‐based model by Ning et al. [21] for diagnosing UM‐EGC based on pathologic images, and the prediction model for curative ESD of UM‐EGC recently developed by Yang et al. [22]. Advancements in artificial intelligence (AI) have also emerged, with Shin et al. [23] employing surface‐enhanced Raman spectroscopy and Gong et al. [24] developing a deep learning‐based clinical decision support system for real‐time endoscopic detection and classification of gastric lesions. These AI‐driven approaches, often integrating endoscopic and histopathological imaging [25], offer promising performance for enhancing diagnostic accuracy and transparency for gastric lesions. Our multivariate analysis systematically evaluated the predictive value of conventional clinicopathological and endoscopic parameters for UM‐EGC, which identified open‐type (O1–O3) atrophic gastritis, flat or depressed morphology, faded appearance, tumor location on the greater curvature or anterior wall, metachronous carcinoma, and previous *H. pylori* eradication as independent predictors of UM‐EGC risk, which was consistent with the data of the National Cancer Database of the United States [26]. Among them, faded appearance, which is the whitish discoloration characteristic of UM‐EGC, likely reflects profound alterations in the tumor microenvironment (TME) and angiogenic dynamics. UM‐EGC, particularly those with poorly cohesive morphology (e.g., signet‐ring cell carcinoma), shows an infiltrative growth pattern that disrupts the normal mucosal microvasculature [27]. This disruption results in reduced microvascular density within the tumor area, leading to mucosal ischemia and the macroscopic appearance of pallor or fading. Furthermore, the TME in UM‐EGC is often characterized by desmoplastic stromal reaction, where abundant collagen deposition, fibroblast activation, and immune cell compositions further compress residual microvessels, exacerbating the avascular state [28, 29]. Recent studies have also highlighted the role of angiogenic switch imbalance [22], shifting the balance toward vascular quiescence or regression. Further studies integrating these TME‐based explanations are warranted to enhance the clinical relevance of our endoscopic findings and bridge the gap between routine observations and tumor biology.

Several studies have investigated the prediction of GC risk, yet most of them focused on the prediction of lymph node metastasis or curative resection in UD‐EGC, rather than the pretreatment endoscopic diagnosis of UD‐EGC or UM‐EGC itself. For example, Guo et al. [30] developed an artificial neural network model to evaluate the risks of lymph node metastasis in patients with non‐intestinal type EGC based on clinicopathological factors, while Mao et al. [31] constructed a deep learning‐based model integrating clinical, pathological, and endoscopic data for the prediction of lymph node metastasis, achieving an AUC of 0.843–0.875. Similarly, Li et al. [32] and You et al. [33] developed nomograms for the risk of lymph node metastasis based on undifferentiated components or signet ring cell histology, respectively, although both were derived from surgically resected specimens and thus were primarily related to postoperative decision‐making. In contrast, tools specifically designed for the pretreatment endoscopic diagnosis of UM‐EGC itself—the critical first step guiding subsequent management—remain limited. Our nomogram fills this gap by providing a simple, accessible tool for pretreatment risk stratification of UM‐EGC. Developed and externally validated in two different campuses of the same hospital system in Beijing, it integrates readily available endoscopic and clinical parameters without costly assays, making it more suitable for resource‐limited settings. Although the training and validation cohorts differed significantly in several baseline characteristics, including lesion multiplicity, macroscopic type, and lesion color, these differences likely stem from real‐world population heterogeneity across the two campuses. This provides a more stringent test of model generalizability. Importantly, our nomogram achieved an AUC of 0.83 (95% CI 0.77–0.87), which offers greater practicality for clinical decision‐making, enabling early identification of high‐risk lesions and personalized management.

There are several limitations to this study. First, this was a single‐center, retrospective study despite that patients were enrolled from two campuses—Xicheng and Tongzhou—of the same hospital system, which draw patients from different catchment areas (central/urban Beijing vs. Tongzhou District of Beijing and the neighboring Hebei Province). This might have introduced selection bias and limited the generalizability of our findings. Therefore, future multi‐center prospective studies with larger and more diverse populations are needed for external validation of our model. We plan to conduct such external validation in collaboration with other institutions to assess the robustness and transportability of our results. Second, the sample size was relatively small, with an EPV of approximately 8, which might have affected the stability of coefficient estimates and limited statistical power for detecting smaller effect sizes. To assess the internal validity, we performed fivefold cross‐validation, which suggested that the model performance was sensitive to the composition of the validation set, highlighting the need for external validation in a larger, more diverse cohort. Despite some variability across folds, the AUC and 95% CI suggest moderate predictive performance and reasonable internal stability. Although a post‐hoc power analysis for the key predictor “faded color” demonstrated a high statistical power (0.91), our study may still be underpowered for the analyses involving less frequent variables or predefined subgroups. Third, we focused exclusively on patients who underwent curative resection for EGC, and thus the characteristics and outcomes of those who had undergone non‐curative resection remain to be explored. Finally, our model was developed using only clinicopathological and endoscopic parameters and did not incorporate molecular biomarkers. Future studies integrating genomic or proteomic data may further improve the predictive accuracy of the model. Prospective studies with pre‐specified sample size calculation and external validation in larger, independent cohorts are warranted to confirm our findings.

## 5 Conclusions

In this study, we identified open‐type atrophic gastritis, flat or depressed morphology, faded color, tumor location on the greater curvature or anterior wall, presence of metachronous carcinoma, and previous *H. pylori* eradication as independent risk factors for UM‐EGC. Based on these factors, we constructed a nomogram with internal and external validation, showing that it may assist endoscopists in the timely and accurate diagnosis of UM‐EGC to facilitate treatment decision‐making in clinical practice.

## Funding

National Key Research and Development Program of China (2022YFC3602101). National Key Research and Development Program of China (2022YFC3602104). Beijing Research Ward Excellence Program (BRWEP2024W162020105).

## Conflicts of Interest

The authors declare no conflicts of interest.

## Supporting information


**Table S1:** Baseline characteristics between the training and validation cohort.

## Data Availability

The data generated and analyzed during this study are not publicly available due to patient privacy and ethical restrictions Scientific Research Ethical Licensing Committee of the Beijing Friendship Hospital, Capital Medical University (2018‐P2‐058‐01). However, they can be requested from the corresponding author upon reasonable request, subject to institutional approval.
