# Supplementary material for: Development and Validation of an Individualized Nomogram to Identify Undifferentiated‐Predominant Mixed‐Type Early Gastric Cancer
Source: J Dig Dis. 2026 Apr 28;27(3-4):118–28. doi: 10.1111/1751-2980.70047 (PMC13281755; doi:10.1111/1751-2980.70047)
Supplement: Supplementary file 1 — Table S1: Baseline characteristics between the training and validation cohort. [file CDD-27-118-s001.docx]

Supplementary Table 1. Baseline characteristics between the training and validation cohort.

| Characteristics (*n*, %) | Overall  (*N* = 577) | Training cohort  (*n* = 493) | Validation  cohort  (*n* = 84) | *p* value |
| --- | --- | --- | --- | --- |
| Age (years) |  |  |  | 0.623 |
| ≤ 60 | 187 (32.4) | 155 (31.4) | 32 (38.1) |  |
| > 60 | 390 (67.6) | 338 (68.6) | 52 (61.9) |  |
| Sex |  |  |  | 0.822 |
| Male | 401 (69.5) | 344 (69.8) | 57 (67.9) |  |
| Female | 176 (30.5) | 149 (30.2) | 27 (32.1) |  |
| Atrophic gastritis |  |  |  | 0.574 |
| No | 62 (10.7) | 51 (10.3) | 11 (13.1) |  |
| Closed type (C1–C3) | 404 (70.0) | 348 (70.6) | 56 (66.7) |  |
| Open type (O1–O3) | 111 (19.2) | 94 (19.1) | 17 (20.2) |  |
| Multiple lesions | 132 (22.9) | 122 (24.7) | 10 (11.9) | 0.014 |
| UM-EGC | 71 (12.3) | 64 (13.0) | 7 (8.3) | 0.308 |
| Macroscopic type |  |  |  | 0.001 |
| IIa | 153 (26.5) | 132 (26.8) | 21 (25.0) |  |
| IIa + IIc | 179 (31.0) | 157 (31.8) | 22 (26.2) |  |
| IIb | 61 (10.6) | 60 (12.2) | 1 (1.2) |  |
| IIc | 184 (31.9) | 144 (29.2) | 40 (47.6) |  |
| White light color, faded | 64 (11.1) | 40 (8.1) | 24 (28.6) | < 0.001 |
| Ulceration | 58 (10.1) | 50 (10.1) | 8 (9.5) | 1.000 |
| Vertical location |  |  |  | 0.278 |
| Upper third | 108 (18.7) | 96 (19.5) | 12 (14.3) |  |
| Middle third | 162 (28.1) | 133 (27.0) | 29 (34.5) |  |
| Lower third | 307 (53.2) | 264 (53.5) | 43 (51.2) |  |
| Horizontal location |  |  |  | 0.231 |
| Lesser curvature | 203 (35.2) | 178 (36.1) | 25 (29.8) |  |
| Greater curvature | 86 (14.9) | 68 (13.8) | 18 (21.4) |  |
| Anterior wall | 110 (19.1) | 92 (18.7) | 18 (21.4) |  |
| Posterior wall | 178 (30.8) | 155 (31.4) | 23 (27.4) |  |
| Depth of invasion |  |  |  | 0.510 |
| Mucosal | 521 (90.3) | 443 (89.9) | 78 (92.9) |  |
| Submucosal | 56 (9.7) | 50 (10.1) | 6 (7.1) |  |
| Metachronous carcinoma | 15 (2.6) | 14 (2.8) | 1 (1.2) | 0.612 |
| Xanthoma | 72 (12.5) | 58 (11.8) | 14 (16.7) | 0.281 |
| Hypertension | 234 (40.6) | 205 (41.6) | 29 (34.5) | 0.272 |
| Diabetes mellitus | 116 (20.1) | 94 (19.1) | 22 (26.2) | 0.174 |
| Hyperlipidemia | 230 (39.9) | 191 (38.7) | 39 (46.4) | 0.227 |
| Smoking | 223 (38.6) | 195 (39.6) | 28 (33.3) | 0.337 |
| Alcohol consumption | 177 (30.7) | 151 (30.6) | 26 (31.0) | 1.000 |
| Family history of gastric cancer | 140 (24.3) | 122 (24.7) | 18 (21.4) | 0.604 |
| *Helicobacter pylori* infection |  |  |  | 0.232 |
| Current | 156 (27.0) | 135 (27.4) | 21 (25.0) |  |
| Eradicated | 191 (33.1) | 162 (32.9) | 29 (34.5) |  |
| Negative | 67 (11.6) | 62 (12.6) | 5 (6.0) |  |
| Unclear | 163 (28.2) | 134 (27.2) | 29 (34.5) |  |

Abbreviation: UM-EGC, undifferentiated-type-predominant mixed-type early gastric cancer.
